# Supplementary material for: Molecular identification of Coxiella burnetii in raw milk samples collected from farm animals in districts Kasur and Lahore of Punjab, Pakistan
Source: PLoS One. 2024 Jun 6;19(6):e0301611. doi: 10.1371/journal.pone.0301611 (PMC11156400; doi:10.1371/journal.pone.0301611)
Supplement: S1 File — (PDF) [file pone.0301611.s001.pdf]

Molecular identification of *Coxiella burnetii* in raw milk samples collected from farm animals in districts Kasur and Lahore of Punjab, Pakistan

Shahpal Shujat, Wasim Shehzad, Aftab Ahmad Anjum, Julia A. Hertl, Yrjö T. Gröhn, Muhammad Yasir Zahoor

SUPPLEMENTARY TABLES

Supplementary Table S1 Univariable analysis of *Coxiella burnetii* (Q Fever) prevalence in 11 villages, districts Kasur and Lahore in Punjab province, Pakistan, 2019. P-values determined by Fisher exact tests of association.

Variable	Category	Q Fever		P value
		Pos. / Tested	Prevalence (%)	
**District**	Lahore	7/136	5.1	0.635
	Kasur	12/168	7.1	
**Village**	Mustafabad, Kasur	0/2	0.0	0.434
	Daftu, Kasur	0/16	0.0	
	Dheng Shah, Kasur	0/23	0.0	
	Fateh Pur, Kasur	2/16	12.5	
	Harbanspura, Lahore	2/67	3.0	
	Korian, Lahore	1/23	4.3	
	Man, Kasur	3/24	12.5	
	Mustafabad lalyani, Kasur	2/28	7.1	
	Rohewal, Kasur	2/36	5.6	
	Soyan, Lahore	4/46	8.7	
	Wadana, Kasur	3/23	13.0	
**Ruminant**	Small	6/65	9.2	0.559
	Large	8/138	5.8	
	Both	5/101	5.0	
**Species**	Cattle	5/90	5.6	0.337
	Buffalo	3/88	3.4	
	Goat	4/60	6.7	
	Sheep	7/66	10.6	
**Breed**	Exotic	2/16	12.5	0.263
	Indigenous	17/288	5.9	
**Farming System**	Commercial	5/123	4.1	0.233
	Household	14/181	7.7	

**Feed pattern**	Stallfed	9/159	5.7	0.547
	Grazing	9/108	8.3	
	Both	1/34	2.9	
**Milking Method**	Hand	19/282	6.7	0.378
	Machine and Hand	0/22	0.0	
**Breeding Method**	Artificial insemination	0/11	0.0	0.482
	Other	6/68	8.8	
	Both	13/225	5.8	
**Farm Disinfection**	Yes	18/248	7.3	0.485
	No	1/38	2.6	
**Deworming Performed**	Yes	19/300	6.3	1.000
	No	0/4	0.0	
**Mastitis**	Yes	1/37	2.7	0.487
	No	18/267	6.7	
**Abortion**	Yes	1/5	20.0	0.277
	No	18/299	6.0	
**Body Condition**	Good	0/5	0.0	1.000
	Average	19/297	6.4	
	Weak	0/2	0.0	

SupplementaryTable S2 Controlling species variable for univariate analyses for *Coxiella burnetii* prevalence in districts Kasur and Lahore in Punjab province, Pakistan, 2019. P-values determined by Fisher exact tests of association.

**Breed**	**Cattle**	**Buffalo**	**Goat**	**Sheep**
**Exotic**				
Infected	12.5% (2/16)	0% (0/0)	0% (0/0)	0% (0/0)
Non Infected	87.5%(14/16)	0% (0/0)	0% (0/0)	0% (0/0)
**Indigenous**				
Infected	4.1% (3/74)	3.4% (3/88)	6.7% (4/60)	10.6% (7/66)
Non Infected	95.9%(71/74)	83.5%(71/85)	93.3%(56/60)	89.4%(59/66)
**P value**	0.22	-	-	-

SupplementaryTable S3 Controlling body condition variable for univariate analyses for *Coxiella burnetii* prevalence in districts Kasur and Lahore in Punjab province, Pakistan, 2019. P-values determined by Fisher exact tests of association.

Body Condition	Infection	Cattle Frequency		Buffalo Frequency		Goat Frequency		Sheep Frequency		Overall Frequency	
Weak	Positive	0	0%	0	0%	0		0		0	0%
	Negative	1	100%	1	100%	0		0		2	100%
	Total	1		1		0		0		2	
Average	Positive	5	7%	3	4%	4	7%	7	11%	19	6%
	Negative	81	93%	82	96%	56	93%	59	89%	278	94%
	Total	86		85		60		66		297	297
Good	Positive	0	0%	0	0%	0		0		0	0%
	Negative	3	100%	2	100%	0		0		5	100%
	Total	3		2		0		0		5	
P value		1.00		1.00		-		-		1.00	

Supplementary Table S4 Controlling mastitis variable for univariate analyses for *Coxiella burnetii* prevalence in districts Kasur and Lahore in Punjab province, Pakistan, 2019. P-values determined by Fisher exact tests of association.

Mastitis	Infection	Cattle Frequency		Buffalo Frequency		Goat Frequency		Sheep Frequency		Overall Frequency	
Yes	Positive	0	0%	1	7%	0	0%	0		1	3%
	Negative	14	100%	14	93%	8	100%	0		36	97%
	Total	14		15		8		0		37	
No	Positive	5	7%	2	3%	4	8%	7	12%	18	7%
	Negative	71	93%	71	97%	48	92%	59	88%	249	93%
	Total	76		73		52		66		267	
P value		1.00		0.43		1.00		-		0.49	
