# Supplementary material for: Molecular identification of Coxiella burnetii in raw milk samples collected from farm animals in districts Kasur and Lahore of Punjab, Pakistan
Source: PLoS One. 2024 Jun 6;19(6):e0301611. doi: 10.1371/journal.pone.0301611 (PMC11156400; doi:10.1371/journal.pone.0301611)
Supplement: Attachment — Submitted filename: PONE-D-23-27370_reviewer.pdf [file pone.0301611.s005.pdf]

PLOS ONE

Molecular identification of Coxiella burnetii in raw milk samples collected from farm animals in districts Kasur and Lahore of Punjab, Pakistan

--Manuscript Draft--

Manuscript Number:	PONE-D-23-27370
Article Type:	Research Article
Full Title:	Molecular identification of Coxiella burnetii in raw milk samples collected from farm animals in districts Kasur and Lahore of Punjab, Pakistan
Short Title:	Molecular identification of Coxiella burnetii in raw milk samples of ruminat source.
Corresponding Author:	Muhammad Yasir Zahoor, PhD<br>University of Veterinary and Animal Sciences<br>Lahore, PAKISTAN
Keywords:	Coxiella burnetii; Q fever; milk consumption; ruminant; molecular diagnosis
Abstract:	Coxiella burnetii is the worldwide zoonotic infectious agent for Q fever in humans and animals. Farm animals are the main reservoirs of C. burnetii infection, which is mainly transmitted via tick bites. In humans, oral, percutaneous, and respiratory routes are the primary sources of infection transmission. The clinical signs vary from flu-like symptoms to endocarditis for humans' acute and chronic Q fever. While it is usually asymptomatic in livestock, abortion, stillbirth, infertility, mastitis, and endometritis are its clinical consequences. Infected farm animals shed C. burnetii in birth products, milk, feces, vaginal mucus, and urine. Milk is an important source of infection among foods of animal origin. This study aimed to determine the prevalence of C. burnetii in milk samples of dairy animals from two districts in Punjab, Pakistan, as it has not been reported there so far. Using a convenient approach, the current study included 304 individual milk samples from different herds of cattle, buffalo, goats, and sheep present on 39 farms in 11 villages in the districts of Kasur and Lahore. PCR targeting the IS1111 gene sequence was used for its detection. Coxiella burnetii DNA was present in 19 of the 304 (6.25%) samples. The distribution was 7.14% and 5.15% in districts Kasur and Lahore, respectively. The results showed the distribution in ruminants as 3.4% in buffalo, 5.6% in cattle, 6.7% in goats, and 10.7% in sheep. From the univariate analysis, the clinical signs of infection i.e. mastitis and abortion was analyzed for the prevalence of Coxiella burnetii. The obtained results showed that the abortion was differed significantly for the prevalence of infection in cattle species (P = 0.03). The obtained sequences were identical to the reported sequence of a local strain in district Sahiwal while showing similarity with French and Indian origin sequences. These findings demonstrated that the prevalence of C. burnetii in raw milk samples deserves more attention from the health care system and veterinary organizations in Kasur and Lahore of Punjab, Pakistan. Future studies should include different districts and human populations, especially professionals working with animals, to estimate the prevalence of C.burnetii.
Order of Authors:	Shahpal Shujat
	Wasim Shehzad, DVM, PhD
	Aftab Ahmad Anjum, PhD
	Julia A. Hertl, MS
	Yrjö T. A. Gröhn, DVM, PhD
	Muhammad Yasir Zahoor, DVM, PhD
Additional Information:	
Question	Response
Financial Disclosure<br><br>Enter a financial disclosure statement that	Funding Statement<br>This study was funded by International Research Support Initiative Program, Higher Education Comission, Pakistan fellowship. Grant :I -8/HEC/HRD/2021/11529 awarded

describes the sources of funding for the work included in this submission. Review the submission guidelines for detailed requirements. View published research articles from *PLOS ONE* for specific examples.<br><br>This statement is required for submission and **will appear in the published article** if the submission is accepted. Please make sure it is accurate.<br><br>**Unfunded studies**<br>Enter: *The author(s) received no specific funding for this work.*<br><br>**Funded studies**<br>Enter a statement with the following details:<br>• Initials of the authors who received each award<br>• Grant numbers awarded to each author<br>• The full name of each funder<br>• URL of each funder website<br>• Did the sponsors or funders play any role in the study design, data collection and analysis, decision to publish, or preparation of the manuscript?<br>• **NO** - Include this sentence at the end of your statement: *The funders had no role in study design, data collection and analysis, decision to publish, or preparation of the manuscript.*<br>• **YES** - Specify the role(s) played.<br><br>* typeset	to student. The funder has no role in study design,, data collection and analysis, decision to publish, or preparation of manuscript.
**Competing Interests**<br><br>Use the instructions below to enter a competing interest statement for this submission. On behalf of all authors, disclose any competing interests that could be perceived to bias this work—acknowledging all financial support and any other relevant financial or non-financial competing interests.<br><br>This statement is required for submission and **will appear in the published article** if the submission is accepted. Please make sure it is accurate and that any funding	Competing interests: The authors have declared that no competing interests exists

Powered by Editorial Manager® and ProduXion Manager® from Aries Systems Corporation

sources listed in your Funding Information later in the submission form are also declared in your Financial Disclosure statement.<br><br>View published research articles from *PLOS ONE* for specific examples.<br><br>**NO authors have competing interests**<br><br>Enter: *The authors have declared that no competing interests exist.*<br><br>**Authors with competing interests**<br><br>Enter competing interest details beginning with this statement:<br><br>*I have read the journal's policy and the authors of this manuscript have the following competing interests: [insert competing interests here]*<br><br>* typeset	
**Ethics Statement**<br><br>Enter an ethics statement for this submission. This statement is required if the study involved:<br><br>• Human participants<br>• Human specimens or tissue<br>• Vertebrate animals or cephalopods<br>• Vertebrate embryos or tissues<br>• Field research<br><br>Write "N/A" if the submission does not require an ethics statement.<br><br>General guidance is provided below. Consult the submission guidelines for detailed instructions. **Make sure that all information entered here is included in the Methods section of the manuscript.**	N/A

**Format for specific study types**<br><br>**Human Subject Research (involving human participants and/or tissue)**<br>• Give the name of the institutional review board or ethics committee that approved the study<br>• Include the approval number and/or a statement indicating approval of this research<br>• Indicate the form of consent obtained (written/oral) or the reason that consent was not obtained (e.g. the data were analyzed anonymously)<br><br>**Animal Research (involving vertebrate animals, embryos or tissues)**<br>• Provide the name of the Institutional Animal Care and Use Committee (IACUC) or other relevant ethics board that reviewed the study protocol, and indicate whether they approved this research or granted a formal waiver of ethical approval<br>• Include an approval number if one was obtained<br>• If the study involved *non-human primates*, add *additional details* about animal welfare and steps taken to ameliorate suffering<br>• If anesthesia, euthanasia, or any kind of animal sacrifice is part of the study, include briefly which substances and/or methods were applied<br><br>**Field Research**<br><br>Include the following details if this study involves the collection of plant, animal, or other materials from a natural setting:<br>• Field permit number<br>• Name of the institution or relevant body that granted permission	
**Data Availability**<br><br>Authors are required to make all data underlying the findings described fully available, without restriction, and from the time of publication. PLOS allows rare exceptions to address legal and ethical concerns. See the PLOS Data Policy and FAQ for detailed information.	Yes - all data are fully available without restriction

A Data Availability Statement describing where the data can be found is required at submission. Your answers to this question constitute the Data Availability Statement and **will be published in the article**, if accepted.<br><br>**Important:** Stating 'data available on request from the author' is not sufficient. If your data are only available upon request, select 'No' for the first question and explain your exceptional situation in the text box.<br><br>Do the authors confirm that all data underlying the findings described in their manuscript are fully available without restriction?	
**Describe where the data may be found in full sentences. If you are copying our sample text, replace any instances of XXX with the appropriate details.**<br><br>• If the data are **held or will be held in a public repository**, include URLs, accession numbers or DOIs. If this information will only be available after acceptance, indicate this by ticking the box below. For example: *All XXX files are available from the XXX database (accession number(s) XXX, XXX.*).<br>• If the data are all contained **within the manuscript and/or Supporting Information files**, enter the following: *All relevant data are within the manuscript and its Supporting Information files.*<br>• If neither of these applies but you are able to provide **details of access elsewhere**, with or without limitations, please do so. For example:<br><br>*Data cannot be shared publicly because of [XXX]. Data are available from the XXX Institutional Data Access / Ethics Committee (contact via XXX) for researchers who meet the criteria for access to confidential data.*<br><br>*The data underlying the results presented in the study are available from (include the name of the third party*	No

and contact information or URL).<br>• This text is appropriate if the data are owned by a third party and authors do not have permission to share the data.<br><br>* typeset	
Additional data availability information:	

Manuscript

Click here to access/download;Manuscript;Manuscript SS2 PLOSONE.docx

Molecular identification of *Coxiella burnetii* in raw milk samples collected from farm animals in districts Kasur and Lahore of Punjab, Pakistan

Shahpal Shujat [1 2], Wasim Shehzad [2], Aftab Ahmad Anjum [3], Julia A. Hertl [2], Yrjö T. Gröhn,[2] Muhammad Yasir Zahoor [1*]

[1]Institute of Biochemistry and Biotechnology, University of Veterinary and Animal Sciences, Lahore, Punjab, Pakistan, [2]Department of Population Medicine and Diagnostic Sciences, College of Veterinary Medicine, Cornell University, Ithaca, New York, United States of America, [3]Institute of Microbiology, University of Veterinary and Animal Sciences, Lahore, Punjab, Pakistan

*yasir.zahoor@uvas.edu.pk

Abstract

Coxiella burnetii is the worldwide zoonotic infectious agent for Q fever in humans and animals. Farm animals are the main reservoirs of *C. burnetii* infection, which is mainly transmitted via tick bites. In humans, oral, percutaneous, and respiratory routes are the primary sources of infection transmission. The clinical signs vary from flu-like symptoms to endocarditis for humans' acute and chronic Q fever. While it is usually asymptomatic in livestock, abortion, stillbirth, infertility, mastitis, and endometritis are its clinical consequences. Infected farm animals shed *C. burnetii* in birth products, milk, feces, vaginal mucus, and urine. Milk is an important source of infection among foods of animal origin. This study aimed to determine the prevalence of *C. burnetii* in milk samples of dairy animals from two districts in Punjab, Pakistan, as it has not been reported there so far. Using a convenient approach, the current study included 304 individual milk samples from different herds of cattle, buffalo, goats, and sheep present on 39 farms in 11 villages in the districts of Kasur and Lahore. PCR targeting the *IS1111* gene sequence was used for its detection. *Coxiella burnetii* DNA was present in 19 of the 304 (6.25%) samples. The distribution was 7.14% and 5.15% in districts Kasur and Lahore, respectively. The results showed the distribution in ruminants as 3.4% in buffalo, 5.6% in cattle, 6.7% in goats, and 10.7% in sheep. From the univariate analysis, the clinical signs of infection i.e. mastitis and abortion was analyzed for the prevalence of *Coxiella burnetii*. The obtained results showed that the abortion was differed significantly for the prevalence of infection in cattle species (P = 0.03). The obtained sequences were identical to the reported sequence of a local strain in district Sahiwal while showing similarity with French and Indian origin sequences. These findings demonstrated that the prevalence of *C. burnetii* in raw milk samples deserves more attention from the health care system and veterinary organizations in Kasur and Lahore of Punjab, Pakistan. Future studies should include different districts and human populations, especially professionals working with animals, to estimate the prevalence of *C.burnetii*.

Keywords: *Coxiella burnetii*, Q fever**,** milk consumption, ruminant, molecular diagnosis

Introduction

Coxiella burnetii is a gram-negative obligate intracellular bacterium. It is an etiological agent of Q fever in humans and animals [1]. The Center for Disease Control and Prevention has classified it as a category B bioweapon due to its aerosolized property and rapid spread. This bacterium can cause disease in a low infectious dose; only 1-10 organisms are required for this infection [2-4]. *Coxiella burnetii*'s spore-like structure can persist under harsh environmental conditions and stress [5].

Coxiella burnetii has been globally ranked among the top 13 priority zoonotic pathogens. It can infect different host species, including domestic, wild, and marine mammals, birds, reptiles, and arthropods. Ruminants are the main reservoirs for this pathogen [6]. It is a tick-borne pathogen; thus, it is transmitted to ruminants mainly through ticks [7]. In humans, infection may occur through inhalation of particles dispersed from environmental dust [8] and direct contact with contagious milk, meat, urine, semen, and feces [9]. Clinical manifestation of this bacterium in

ruminants includes stillbirth, abortion, mastitis, endometritis, and other reproductive disorders [10, 11]. In humans, flu, fever, hepatitis, and endocarditis are the main manifestations [12].

Coxiella burnetii was first identified in Australian abattoir workers [13], has been considered endemic, and has a worldwide distribution, including Pakistan. It has gained international attention since the outbreak in the Netherlands from 2007 to 2010, and has affected humans and farm animals in other European countries [11]. Most cases remain undiagnosed due to a lack of proper diagnostic tools in developing countries like Pakistan.

Coxiella burnetii has been detected using serological and molecular tests from numerous samples, i.e., blood, serum, milk, and meat. Culturing techniques for *C. burnetii* detection are rarely used because of its high pathogenicity. PCR is the most sensitive molecular technique for the detection of *C. burnetii.* Single copy and Multicopy gene targets are used for its detection. The superoxide dismutase gene and *IS1111* gene are single and multicopy gene targets [14].

Coxiella burnetii is a neglected pathogen in Pakistan, although it strongly impacts an infected country's economy and public health. Appropriate farm management and public awareness are required to control this infection. Moreover, the infection remains largely undetected mainly due to limited diagnostic facilities and the lack of sufficient training to healthcare workers and clinical physicians for this contagious disease in developing countries like Pakistan. Notably, in the previous 65 years, there have been only about six publications on human and animal Q fever from Pakistan in the international databank [15- 20]. Information regarding *C. burnetii*'s manifestation in milk obtained from small and large ruminants for human consumption has not been assessed so far. The objective of the current study was to estimate the prevalence of *C. burnetii* in milk samples collected from ruminants used for human consumption.

Methodology

Study area and Sampling

The Advanced Studies and Research Board at the University *of Veterinary and Animal Sciences* in Lahore, Pakistan approved this study in its 50th meeting held on 8 -02- 2019. The sampling was conducted in 2019 with the help of the livestock department of Districts Kasur and Lahore. The samples were collected using a convenient approach. The study included 304 individual milk samples of cattle, buffalo, goat, and sheep collected from 39 farms in 11 Kasur and Lahore villages. Milk samples of about 5 ml were collected in 15 ml sterile falcon tubes, and the information was recorded in the sample collection data book. The collected samples were transported at 4 to 8 °C temperature.

Milk Processing and DNA isolation

Milk samples were stored at -20 °C temperature and further processed for DNA extraction using a manual method. Milk samples of 200 µl were placed in a microcentrifuge tube and centrifuged at 14500 rpm for 15 min at 4 °C, and the cream layer was separated [22]. Lysis buffer of 700 µl and 10 µl of proteinase K were taken in its pellet and incubated at 56 °C overnight. After overnight incubation, 500 µl of PCI was added and vortexed until the solution turned milky. Then it was centrifuged under identical conditions as mentioned above. Three layers were

formed, and the upper transparent layer containing DNA was taken into a separate microcentrifuge tube. Two parts of isopropanol and 200 µl of chilled absolute ethanol were added in 1 part aqueous transparent layer and incubated for 20 min at -20°C. It was then centrifuged under the same conditions, the supernatant was discarded, and the pellet was taken. The taken pellet was washed using 200 µl of 70% ethanol and centrifuged under the same conditions. Then the supernatant was discarded, leaving the pellet drying overnight to evaporate ethanol, and acting as a PCR inhibitor. The dried pellet was dissolved in 20 µl of distilled water in a water bath and heat shocked at 70°C for 40 minutes. DNA quality and quantity were assessed using a spectrophotometer.

Molecular assay and sequence analysis

Coxiella burnetii was diagnosed using multiple copy gene amplification assays targeting transposase gene, i.e., *IS1111*; specific primers were used for this assay. The set of primers used for the PCR amplification assay was sequenced as 5'-GTCTTAAGGTGGGCTGCGTG-3'and 5`-CCCCGAATCTCATTGATCAGC-3` for forward and reverse primer [23]. The diagnostic assay was validated using Vircell Amplirun® Coxiella DNA Control. Each PCR reaction test contained 12.5 µl of 2X master mix, 1.25 µl of 10µM forward and reverse primer, and 1µl of 50- 100 ng DNA in a final volume up to 25 µl by adding nuclease-free water. PCR reaction was performed using a 96 well Applied Biosystems by Thermo Fisher Scientific thermal cycler. The conditions for reaction were optimized as initial denaturation at 95 °C for 5 min, final denaturation at 94 °C for 30 sec, annealing at 60 °C for 30 sec, extension at 72 °C for 1 min, repeat steps 1 to 3 for 30 cycles, and final extension at 72 °C for 10 min. PCR products were analyzed on 2% agarose gel, and specific product was identified, i.e., 294 bp was observed during analysis. The positive samples were sequenced from commercially available services. The sequences were analyzed for phylogenetics using the MEGA version 6.0 bioinformatics tool. Alignment and phylogenetic tree construction of 12 sequences, including two query sequences, were performed using the MEGA tool by the maximum likelihood method [24].

Data Analysis

The data were recorded in a Microsoft Excel spreadsheet. The analysis was performed using SAS 9.4 statistical package. Chi-square tests were performed in PROC FREQ, and logistic regression models were fitted (PROC LOGISTIC), with the presence/absence of *C. burnetii* DNA as the outcome.

Ethics statement

The University ethically approved the Veterinary and Animal Sciences Advanced Studies and Research Board study in its 50th meeting held on 8 -02- 2019.

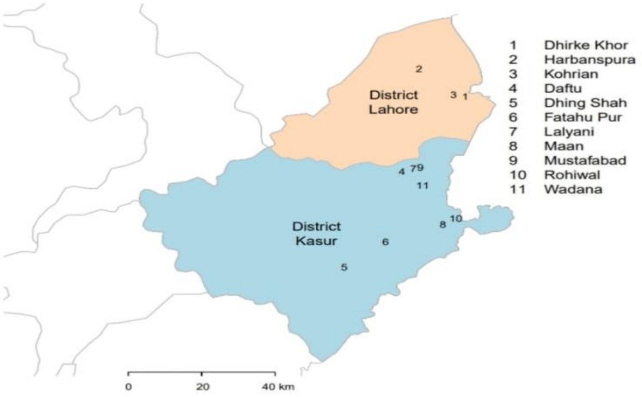

Fig.1. A map showing the sampling locations in the study area of districts Kasur and Lahure, Punjab.

Results

Of 304 samples, 19 (6.25%) were positive for *C. burnetii* DNA by PCR detection using the *IS1111* sequence. The observed results showed a higher prevalence of *C. burnetii* in district Kasur (7.14%) when compared with district Lahore (5.15) (Table 1), although it was not statistically significant. *Coxiella burnetii* prevalence was distributed in four species of ruminants: 3.4% in buffalo, 5.6% in cattle, 6.7% in goats, and 10.6% in sheep (Table 1). Thus, the prevalence of *C. burnetii* was higher in milk samples obtained from goats and sheep than in cattle and buffalo, although the difference was not statistically different (Table 2). From a logistic regression model with district, species, and an interaction between district and species, in Lahore, sheep were 13 times (95% confidence limits: 1.36, 126.02) more likely to test positive than were buffalo, and were 10.4 times (95% confidence limits: 1.07, 100.0) more likely to test positive than were cattle. When abortion factor was analyzed, the prevalence was statistically differed in cattle. (Table 3).

When the sequences were aligned on the basis of their origin, the results showed that in the current study, the sequences obtained from district Lahore were clustered with previously reported sequences from districts Lahore and Sahiwal because of their close geographical proximity. At the same time, the sequences obtained from district Kasur were clustered separately with those obtained from India and France (Fig 1).

Table 1. Prevalence of *Coxiella burnetii* in milk samples collected from different species by PCR during 2019, from districts Lahore and Kasur, Pakistan

	Number of examined samples	Number of positive samples	Percentage
**Species**			
Cattle	90	5	5.6
Buffalo	88	3	3.4
Goat	60	4	6.7
Sheep	66	7	10.6
**District**			
Lahore	136	7	5.2
Kasur	168	12	7.1
Total	304	19	6.25

Table 2. Prevalence of *Coxiella burnetii* in milk samples collected during 2019 from different species and its distribution in districts Lahore and Kasur, Pakistan

Species	District Lahore	District Kasur	Overall	P Value
Cattle	2.5% (1/40)	8% (4/50)	5.6% (5/90)	0.25
Buffalo	2% (1/50)	5.3% (2/38)	3.4% (3/88)	0.40
Goat	3.7% (1/27)	9.1% (3/33)	6.7% (4/60)	0.40
Sheep	21.1% (4/19)	6.4% (3/47)	10.6% (7/66)	0.07

Table 3. Prevalence of *Coxiella burnetii* in milk samples collected from different species when the clinical sign, abortion was analyzed using univariate analysis.

**Abortion**	**Cattle**	**Buffalo**	**Goat**	**Sheep**
**Yes**				
Infected	20% (1/5)	0% (0/3)	0% (0/4)	0% (0/7)
Non Infected	2.4% (2/85)	0% (0/85)	3.6% (2/56)	0% (0/59)
**No**				
Infected	80% (4/5)	100% (3/3)	100% (4/4)	100% (7/7)
Non Infected	97.6% (83/85)	100% (85/85)	96.4% (54/56)	100% (59/59)
**P value**	**0.03**	Row column sum is 0	0.70	Row column sum is zero

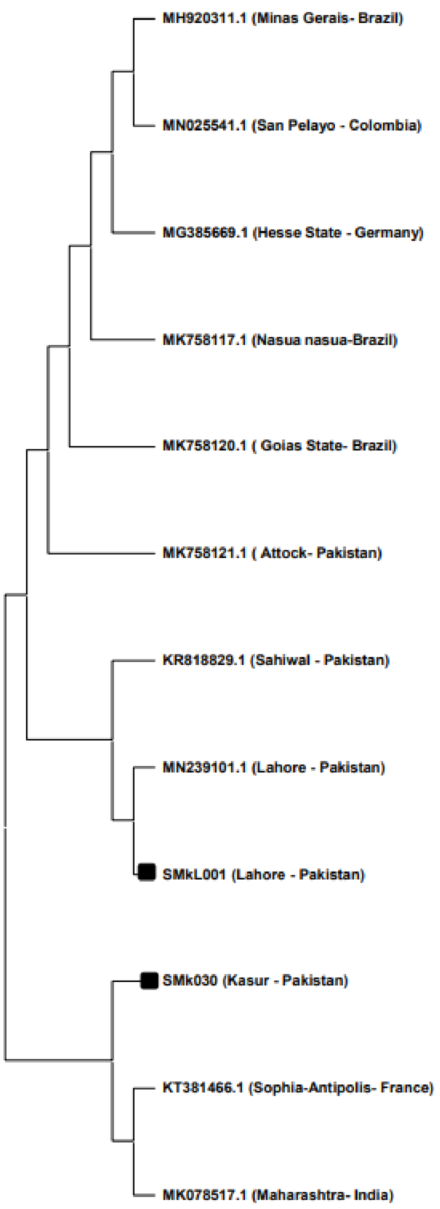

Fig. 2 The phylogenetic relationship of *C.burnetii IS1111* gene sequence recovered from milk samples in 2019 in districts Lahore and Kasur.Labeled sequences are query sequences.

Discussion

The aim of the present study was molecular detection and characterization of *C. burnetii* DNA in raw milk samples collected from different herds of ruminants in the districts of Lahore and Kasur, Pakistan. The first evidence of *C. burnetii* DNA in milk was obtained during this study in Pakistan. Previous studies evaluated the pathogen's presence in meat ,ticks, blood, and serum samples collected from ruminants, environmental samples (i.e., soil samples obtained from animal farms), and human blood samples [15 -21]. *Coxiella burnetii* in raw milk from ruminants was not studied previously. The absence of previous records was due to unawareness and neglect of the disease caused by this pathogen. However, *C. burnetii* is a zoonotic pathogen with worldwide distribution (Maurin & Raoult, 1999). It has been known since the 1930s when it was first reported by Australian abattoir workers [13]. Ticks are considered as main reservoirs for *Coxiella burnetii* and are responsible for the transmission of this pathogen to ruminants.The signs and symptoms observed in ruminants include stillbirth, abortion, mastitis, endometritis, and other reproductive disorders [11, 12]. In humans, flu, fever and hepatitis are observed symptoms in acute Q fever while endocartitis may occur in chronic cases [12]. In humans, this infection can occur through inhalation of particles dispersed from environmental dust and direct contact with the contagious milk, meat, urine, semen, and feces [9]. The current study was based on a one health approach and this zoonotic pathogen was recovered from milk samples used for human consumption. The estimated overall prevalence of *C. burnetii* was 6.25%, while it was 7.14% in district Kasur and 5.15% in district Lahore. Previous studies suggested a 36.87% *C. burnetii* prevalence in blood samples of small ruminants from district Kasur while the prevalence of *C. burnetii* was estimated at 32.1% and 12.5% in cattle and buffaloes, respectively [25, 26]. For district Lahore, the prevalence of infection was 4.8% in environmental samples from a previously reported study [19].

Based on the results of this study, *C. burnetii* was detected in 6.7% of goat milk samples by PCR using the *IS1111* gene sequence. The prevalence of *C. burnetii* in districts Lahore and Kasur was 2% and 9%, respectively. The previous study estimated the prevalence of infection in goat blood samples as 30% in district Kasur [25]. The reported prevalence of infection in other countries was 6.3 – 12.1% in Belgium [27], 14.3% in the USA [28], and 17.2% in Lebanon [29]. Goat's milk is frequently consumed in various countries throughout the world. Therefore, goat milk used for human consumption should be screened for *C. burnetii*, among other pathogens.

The results showed that 10.6% of sheep milk samples were positive for the causative agent of Q fever. The distribution in the two districts was 21.1% and 6.4% in Lahore and Kasur, respectively. In another recent study, *C. burnetii* prevalence was reported as 46.9% in the Kasur district [26]. The prevalence of *C. burnetii* in other countries was 10% in Lebanon [28], 4% in Hungary [30], 6.5% in Turkey [31], and 22% in Spain [32]. According to the findings of this study and previous studies in Pakistan and other countries, it seems that *C. burnetii* is common in sheep. Like goat milk, there is a strong interest in consuming raw sheep's milk and its products in Pakistan, especially in rural and nomadic populations that consume milk from these species. Therefore, paying attention to milk-borne pathogens in such communities is essential, and

veterinary organizations must prioritize control and prevention strategies in livestock. The current study estimated *C. burnetii* prevalence in cattle as 5.6%; the prevalence was 2.5% in Lahore district and 8% in Kasur district. The prevalence of *C. burnetii* in buffaloes was 3.4%. The distribution of *C. burnetii* prevalence in buffaloes in Lahore and Kasur was estimated at 2% and 5.3%, respectively. A previous study showed 32.12% and 12.5% in cattle and buffaloes for the prevalence of *C. burnetii* in district Kasur [25]. Different prevalences of *C. burnetii* have been reported in cattle milk from other countries: 8.7% in Hungary [30], 15.1% in Lebanon [29], 18.8% in the Netherlands [33] and 27% in Italy [34]. Therefore, shedding of pathogens in milk by bovines appears to be the most critical route of spreading this bacterium in the environment in all investigated countries. Future studies should include other districts of Punjab, Pakistan, and sectors of the human population at risk, especially professionals, i.e., farm workers and veterinarians. In this study, molecular evidence of *C. burnetii* was detected in milk samples of dairy animals in the districts of Kasur and Lahore. These findings demonstrated that *C. burnetii* prevalence, especially in raw milk samples, could pose a severe risk of Q fever to farm workers and consumers in Punjab, Pakistan.

Conclusion

Molecular evidence of *C. burnetii* was observed in milk samples of cattle, buffalo, goats, and sheep collected from the farm animals in two districts of Punjab, Pakistan. These findings emphasized that the prevalence of *C. burnetii*, especially in raw milk samples, deserves more attention from the health care system and dairy industry in Kasur and Lahore of Punjab, Pakistan. Future studies must include other districts and risk evaluation in the human population for the infection, especially in professionals, i.e., farm workers and veterinarians.

Data Availability Statement

The original contribution presented in the study are included in the article/Supplementary Materials, further inquiries can be directed to the corresponding author.

Funding Statement

This study was funded by International Research Support Initiative Program, Higher Education Comission, Pakistan fellowship. Grant :I -8/HEC/HRD/2021/11529 awarded to student. The funder has no role in study design,, data collection and analysis, decision to publish, or preparation of manuscript.

Author Contributions

SS is a PhD student and the research article data are a part of her thesis dissertation. WS helped in sample collection. AAA helped in data collection. JAH helped in data analysis. YTG helped in data analysis, interpretation and write up as co –supervisor. MYZ supervised the study.

Acknowledgements

The authors are grateful to Assistant Director Livestock District Kasur Dr. Musarat, Veterinary assistants in Livestock Department District Kasur, farmers and Assistant Director Livestock, District Lahore, and Veterinary Assistants Livestock Department, District Lahore for assisting in sample collection and data recording.

Conflict of Interest

The authors declare no conflicts of interest.

Appendix Supplementary Material

Supplementary Table S1. Univariable analysis of *Coxiella burnetii* (Q Fever) prevalence in 11 villages, districts Kasur and Lahore in Punjab province, Pakistan, 2019
SupplementaryTable S2. Controlling species variable for univariate analyses for *Coxiella burnetii* prevalence in districts Kasur and Lahore in Punjab province, Pakistan, 2019
SupplementaryTable S3. Controlling body condition variable for univariate analyses for *Coxiella burnetii* prevalence in districts Kasur and Lahore in Punjab province, Pakistan, 2019
Supplementary Table S4. Controlling mastitis variable for univariate analyses for *Coxiella burnetii* prevalence in districts Kasur and Lahore in Punjab province, Pakistan, 2019
Supplementary Table S5. Controlling abortion variable for univariate analyses for *Coxiella burnetii* prevalence in districts Kasur and Lahore in Punjab province, Pakistan, 2019

References

1. Maurin M, Raoult Df. Q fever. Clinical microbiology reviews. 1999;12(4):518-53.
2. Benenson A, Tigertt W. Studies on Q fever in man. Transactions of the Association of American Physicians. 1956;69:98.
3. Gonder JC, Kishimoto RA, Kastello MD, Pedersen Jr CE, Larson EW. Cynomolgus monkey model for experimental Q fever infection. Journal of Infectious Diseases. 1979;139(2):191-6.
4. Marrie TJ, Langille D, Papukna V, Yates L. Truckin'pneumonia—an outbreak of Q fever in a truck repair plant probably due to aerosols from clothing contaminated by contact with newborn kittens. Epidemiology & Infection. 1989;102(1):119-27.
5. Kersh GJ, Fitzpatrick KA, Self JS, Priestley RA, Kelly AJ, Lash RR, et al. Presence and persistence of Coxiella burnetii in the environments of goat farms associated with a Q fever outbreak. Applied and environmental microbiology. 2013;79(5):1697-703.
6. Eldin C, Melenotte C, Mediannikov O, Ghigo E, Million M, Edouard S, et al. From Q fever to Coxiella burnetii infection: a paradigm change. Clinical microbiology reviews. 2017;30(1):115-90.
7. Stoker M, Marmion B. The spread of Q fever from animals to man: the natural history of a rickettsial disease. Bulletin of the World Health Organization. 1955;13(5):781.
8. Marrero M, Raoult D. Centrifugation-shell vial technique for rapid detection of Mediterranean spotted fever rickettsia in blood culture. The American journal of tropical medicine and hygiene. 1989;40(2):197-9.
9. Johnson JE, Kadull PJ. Laboratory-acquired Q fever: a report of fifty cases. The American journal of medicine. 1966;41(3):391-403.

10. Marrie TJ. 20 Epidemiology of Q Fever. Rickettsial diseases. 2007:281.
11. Arricau-Bouvery N, Rodolakis A. Is Q fever an emerging or re-emerging zoonosis? Veterinary research. 2005;36(3):327-49.
12. Marrie TJ, Stein A, Janigan D, Raoult D. Route of infection determines the clinical manifestations of acute Q fever. Journal of Infectious Diseases. 1996;173(2):484-7.
13. Derrick E. " Q" Fever, a New Fever Entity: Clinical Features, Diagnosis and Laboratory Investigation. Medical Journal of Australia. 1937;2(8).
14. Fenollar F, Raoult D. Molecular genetic methods for the diagnosis of fastidious microorganisms. Apmis. 2004;112(11- 12):785-807.
15. Shujat S, Shehzad W, Anjum AA, Hertl JA, Zahoor MY, Gröhn YT.Molecular detection of Coxiella burnetii in raw meat samples collected from different abattoirs in districts Kasur and Lahore of Punjab, Pakistan. PLoS One. 2023;18(8): e0289944.
16. Ullah Q, Jamil H, Qureshi ZI, Saqib M, Neubauer H. Sero-Epidemiology of Q Fever (Coxiellosis) in Small Ruminants Kept at Government Livestock Farms of Punjab, Pakistan. Pakistan Journal of Zoology. 2019;51(1).
17. Ahmed IP. A serological investigation of Q fever in Pakistan. J Pak Med Assoc. 1987;37:126-9.
18. Ayaz M, Bari A, Humayun A, editors. Coxiellosis in man and animals in northern parts of Pakistan. Proceedings of Pakistan Congress of Zoology; 1993.
19. Kaplan MM, Bertagna P. The geographical distribution of Q fever. Bulletin of the World Health Organization. 1955;13(5):829.
20. Shabbir MZ, Akram S, ul Hassan Z, Hanif K, Rabbani M, Muhammad J, et al. Evidence of Coxiella burnetii in Punjab province, Pakistan. Acta tropica. 2016;163:61-9.
21. Zahid MU, Hussain MH, Saqib M, Neubauer H, Abbas G, Khan I, et al. Seroprevalence of Q fever (Coxiellosis) in small ruminants of two districts in Punjab, Pakistan. Vector-Borne and Zoonotic Diseases. 2016;16(7):449-54.
22. Berri M, Laroucau K, Rodolakis A. The detection of Coxiella burnetii from ovine genital swabs, milk and fecal samples by the use of a single touchdown polymerase chain reaction. Veterinary microbiology. 2000;72(3-4):285-93.
23. Tozer S, Lambert S, Strong C, Field H, Sloots T, Nissen M. Potential Animal and Environmental Sources of Q Fever Infection for Humans in Q ueensland. Zoonoses and public health. 2014;61(2):105-12.
24. Tamura K, Stecher G, Peterson D, Filipski A, Kumar S. MEGA6: molecular evolutionary genetics analysis version 6.0. Molecular biology and evolution. 2013;30(12):2725-9.
25. Iqbal MZ, Durrani AZ, Khan JA, Ahmad N, Usman M, Jabbar A, et al. Molecular epidemiology of Coxiella Brunetii in small ruminants in Punjab, Pakistan: a novel reporting analytical cross sectional study. Tropical Animal Health and Production. 2021;53(1):1-8.
26. Iqbal MZ, Durrani AZ, Khan JA, Ahmad N, Usman M, Jabbar A, et al. Molecular Identification of Coxiella burnetii, and Incidence and Risk Factors of Coxiellosis in Bovines of Punjab, Pakistan. 2021.
27. Boarbi S, Mori M, Rousset E, Sidi-Boumedine K, Van Esbroeck M, Fretin D. Prevalence and molecular typing of Coxiella burnetii in bulk tank milk in Belgian dairy goats, 2009–2013. Veterinary microbiology. 2014;170(1-2):117-24.
28. Loftis AD, Priestley RA, Massung RF. Detection of Coxiella burnetii in commercially available raw milk from the United States. Foodborne pathogens and disease. 2010;7(12):1453-6.

29. Dabaja M, Greco G, Villari S, Vesco G, Bayan A, El Bazzal B, et al. Occurrence and risk factors of Coxiella burnetii in domestic ruminants in Lebanon. Comparative immunology, microbiology and infectious diseases. 2019;64:109-16.
30. Gyuranecz M, Dénes B, Hornok S, Kovács P, Horváth G, Jurkovich V, et al. Prevalence of Coxiella burnetii in Hungary: screening of dairy cows, sheep, commercial milk samples, and ticks. Vector-Borne and Zoonotic Diseases. 2012;12(8):650-3.
31. Öngör H, Cetinkaya B, Karahan M, Acik MN, Bulut H, Muz A. Detection of Coxiella burnetii by immunomagnetic separation-PCR in the milk of sheep in Turkey. Veterinary record. 2004;154(18):570-2.
32. García-Pérez A, Astobiza I, Barandika J, Atxaerandio R, Hurtado A, Juste R. Investigation of Coxiella burnetii occurrence in dairy sheep flocks by bulk-tank milk analysis and antibody level determination. Journal of dairy science. 2009;92(4):1581-4.
33. Van Engelen E, Schotten N, Schimmer B, Hautvast J, Van Schaik G, Van Duijnhoven Y. Prevalence and risk factors for Coxiella burnetii (Q fever) in Dutch dairy cattle herds based on bulk tank milk testing. Preventive Veterinary Medicine. 2014;117(1):103-9.
34. Petruzzelli A, Amagliani G, Micci E, Foglini M, Di Renzo E, Brandi G, et al. Prevalence assessment of Coxiella burnetii and verocytotoxin-producing Escherichia coli in bovine raw milk through molecular identification. Food control. 2013;32(2):532-6.

Click here to access/download
Supporting Information
Supplementary table.docx
