# Supplementary material for: Molecular identification of Coxiella burnetii in raw milk samples collected from farm animals in districts Kasur and Lahore of Punjab, Pakistan
Source: PLoS One. 2024 Jun 6;19(6):e0301611. doi: 10.1371/journal.pone.0301611 (PMC11156400; doi:10.1371/journal.pone.0301611)
Supplement: Attachment — Submitted filename: PONE review.docx [file pone.0301611.s006.docx]

While the study addresses an important public health concern, there are several shortcomings and scientific mistakes that need attention.

**Introduction:**

The introduction is informative, but it lacks a clear statement of the research gap and a hypothesis. The authors could improve the introduction by providing more context on the global prevalence of *C. burnetii* in raw milk and its impact on public health. Furthermore, the reference citations in the introduction are somewhat outdated, and more recent literature should be incorporated to support the study's rationale.

**Methodology:**

The methodology section lacks details on the inclusion/exclusion criteria for farms and animals, potentially introducing selection bias. The use of a convenient sampling approach may not be representative of the overall population, and the authors should discuss the potential limitations associated with this sampling strategy. Additionally, the absence of information on the sensitivity and specificity of the PCR assay used raises concerns about the reliability of the results.

**Discussion:**

While the discussion compares the study findings with previous research, it fails to discuss the implications of the results for public health and the dairy industry in Punjab. Additionally, the discussion could benefit from a more critical analysis of the study limitations, such as the potential for contamination during sample collection and the use of a single gene target for detection.

**Conclusion:**

The conclusion provides a succinct summary, but it lacks specific recommendations for future research or practical implications for stakeholders. A more detailed discussion of the policy and public health implications of the study results would enhance the conclusion.

**Overall, the manuscript should undergo thorough proofreading and editing for language and grammar issues. Additionally, the authors should consider updating references to include more recent publications in the field. Lastly, the manuscript would benefit from a more explicit statement on ethical considerations, especially regarding animal welfare and informed consent for sample collection.**
